# Supplementary figures and images for: Individual consistency in the behaviors of newly-settled reef fish
Source: PeerJ. 2015 May 14;3:e961. doi: 10.7717/peerj.961 (PMC4435502; doi:10.7717/peerj.961)

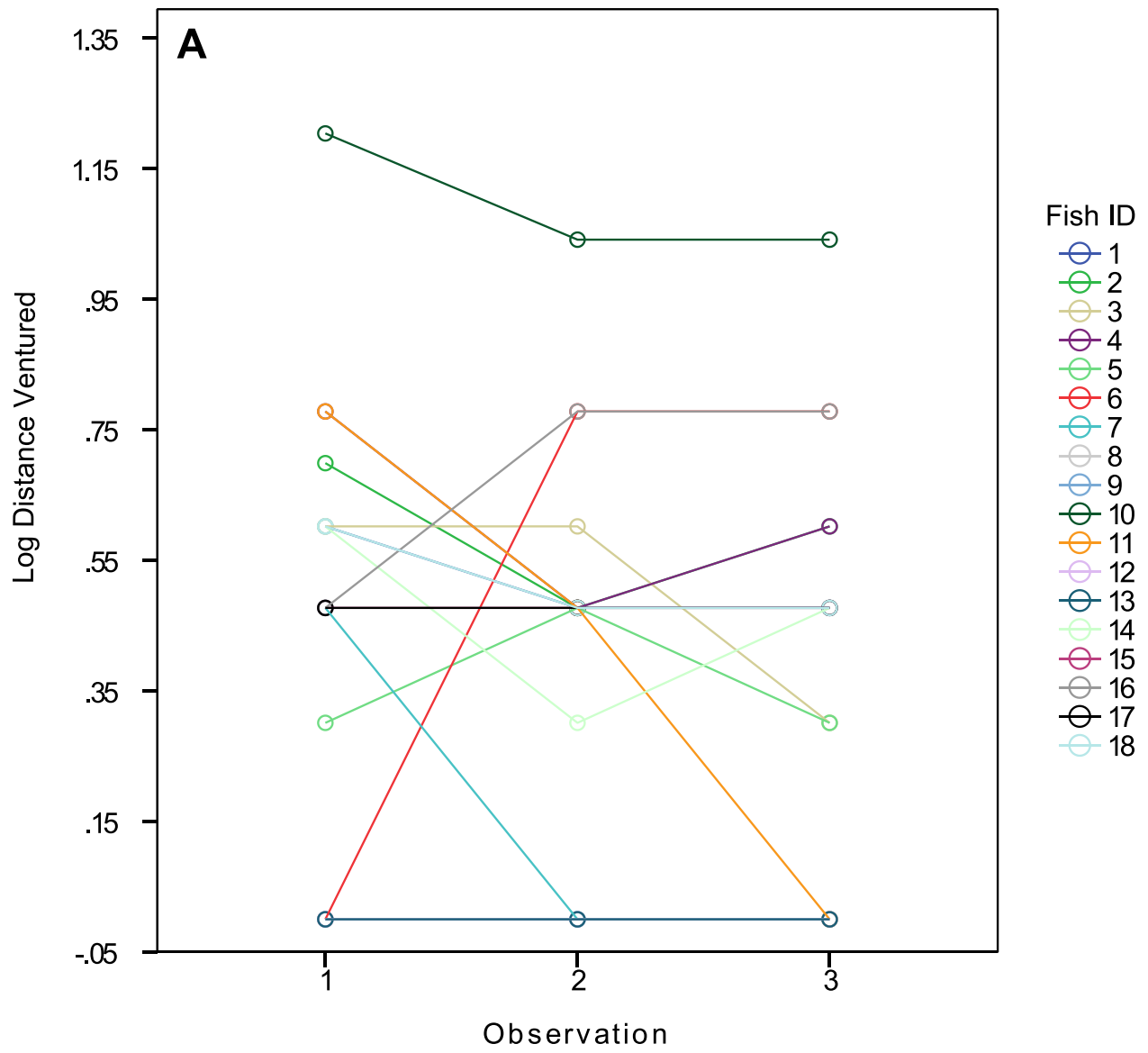

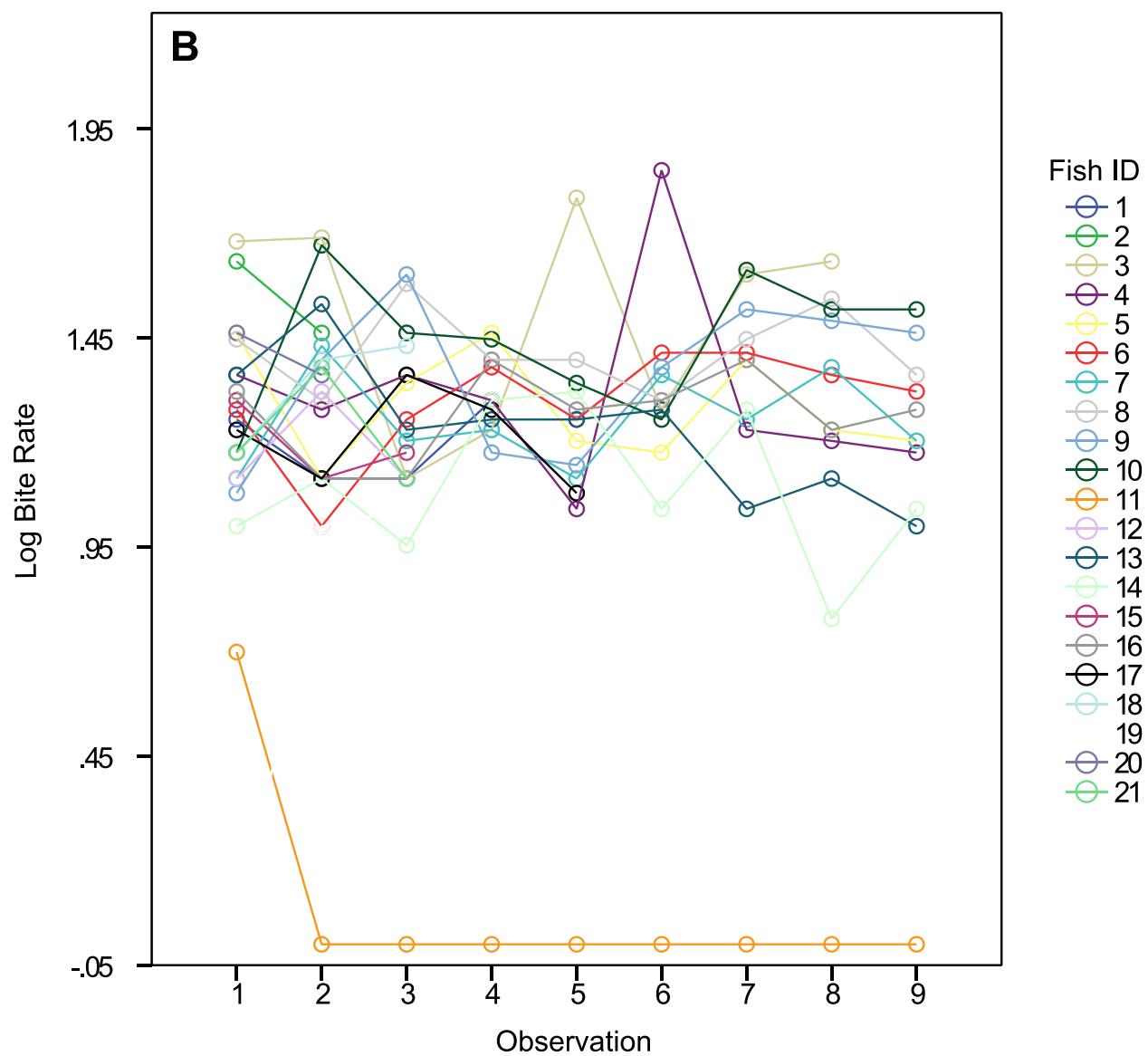

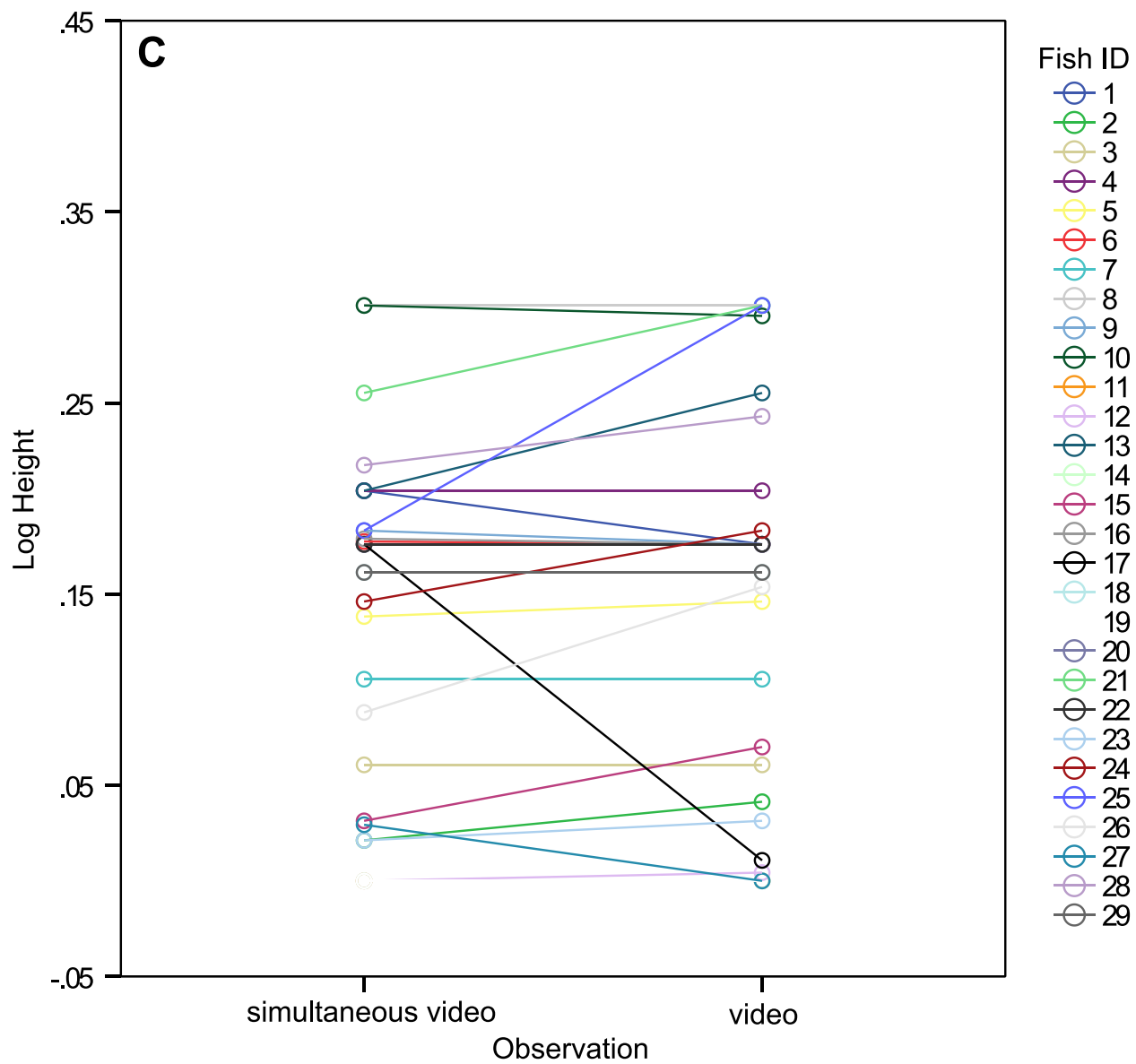

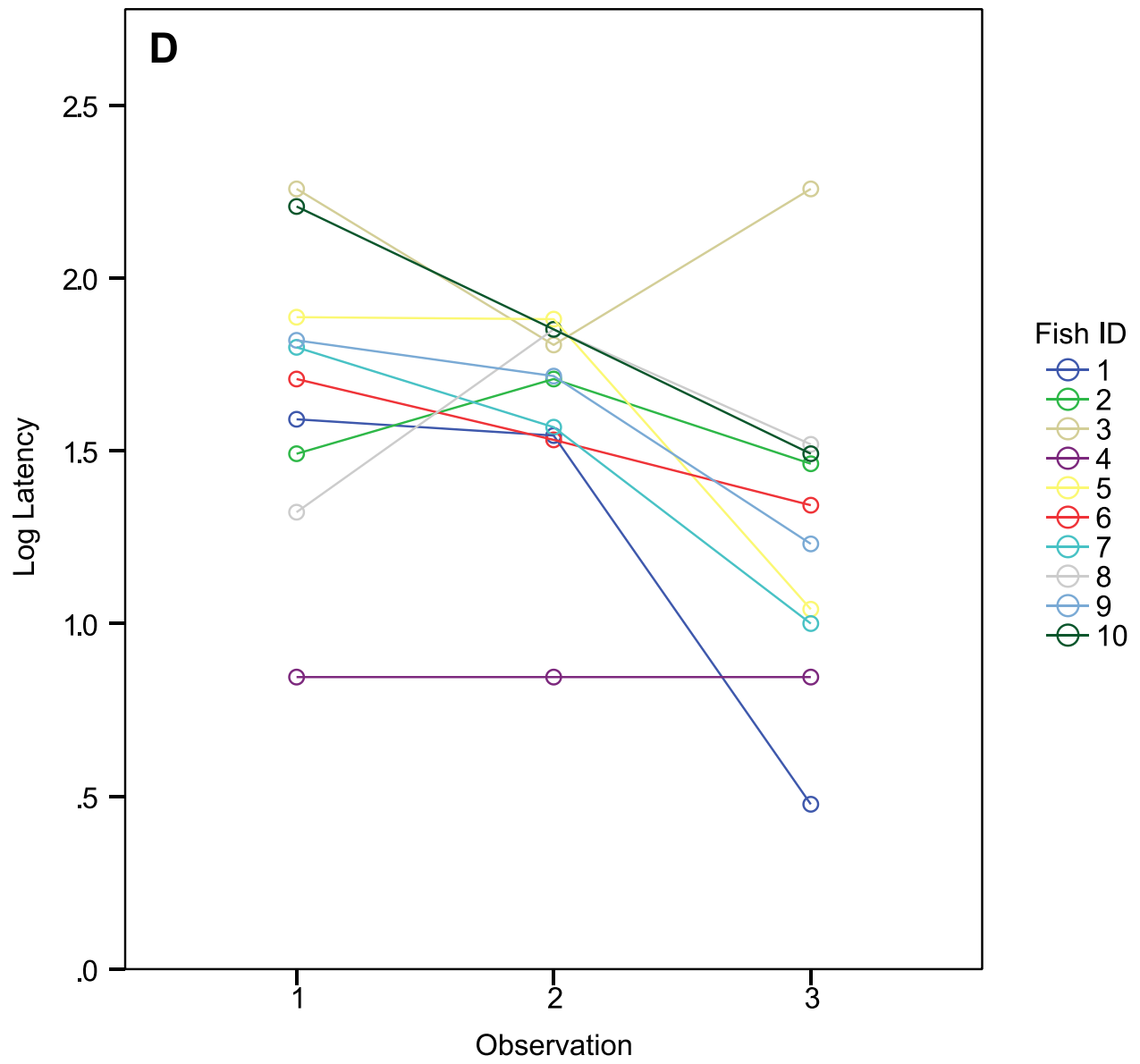

Supplement: Figure S1 — (A) Log transformed distance ventured scores across 3 short term observations in the field. (B) Log transformed bite rate scores across 9 observations in the field over 3 days. (C) Log transformed height scores across 2 video observations in the field. (D) Log transformed latency to emerge scores across 3 short term observations in the laboratory. [file peerj-03-961-s002.pdf]
